# A Systematic Review and Activation Likelihood Estimation Meta-Analysis of fMRI Studies on Sweet Taste in Humans

**DOI:** 10.1093/jn/nxaa071

**Published:** 2020-04-09

**Authors:** Carl A Roberts, Timo Giesbrecht, Nicholas Fallon, Anna Thomas, David J Mela, Tim C Kirkham

**Affiliations:** 1 Department of Psychological Sciences, University of Liverpool, Liverpool, United Kingdom; 2 Unilever R&D, Port Sunlight, United Kingdom; 3 Unilever R&D, Vlaardingen, Netherlands

**Keywords:** sugars, meta-analysis, fMRI, reward, sweet taste

## Abstract

**Background:**

The reward value of palatable foods is often cited as an important influence on eating behaviors, including intake of sugars. However, human neuroimaging studies have generated conflicting evidence on the basic neural representation of taste and reward responses to caloric sweeteners (sucrose and glucose), and most relevant studies have used small subject numbers.

**Objective:**

We conducted a systematic review and a coordinate-based meta-analysis of studies reporting brain responses to oral sugar solutions.

**Methods:**

A systematic search of MEDLINE, Scopus, and PsycINFO through October 2019 identified fMRI studies (in healthy human adults, including those with overweight or obesity) assessing differences in responses to purified sweet and nonsweet taste stimuli. Data were extracted with the primary objective of quantifying evidence for the activation of brain regions associated with caloric sweet taste sensation. We used activation likelihood estimation meta-analysis methods. We also performed multiple sensitivity analyses to assess the generality of effects.

**Results:**

Of 455 unique articles, 15 met the criteria for inclusion. These contributed to 2 primary meta-analyses: *1*) sucrose (13 experiments, 179 coordinates, *n* = 241) and *2*) sucrose + glucose (16 experiments, 209 coordinates, *n* = 262). Consistent activation was apparent in primary taste areas: insula (69.2% of studies) and opercular cortex (76.9% of studies), precentral gyri (53.9% of studies), and globus pallidus and postcentral gyrus (30.8% of studies for each). Evidence of reward activity (caudate) was seen in the primary analyses (30.8% of studies) but not in sensitivity analysis.

**Conclusions:**

We confirm the importance of primary taste areas for gustatory processing in human adults. We also provide tentative evidence for reward-related caudate activity in relation to the sweet taste of caloric sugars. A number of factors affect the observation and interpretation of brain responses, including reward-related activity. Firm conclusions require confirmation with large data set studies.

## Introduction

Intake of free sugars is associated with increased risk of weight gain (1, 2), and sweetness is directly related to orosensory pleasure (3). The reward value of palatable food stimuli, such as sugars, is often proposed as an important mechanism underpinning their influence on eating behaviors and metabolic responses (3, 4). For these reasons, considerable research has been dedicated to assessing the impact of sugars on the neural pathways that mediate reward and the role of reward-motivated eating in food choice and obesity (4). Appetite involves the complex interplay between physiological and psychological mechanisms (5, 6), but hedonic factors alone—such as the sweet taste of energy-rich, sugar-containing foods and beverages—can override any inhibitory influences, drive the desire to eat, and promote energy consumption beyond our metabolic needs (7).

Taste is a basic sense and an integral part of a gustatory system that enables the evaluation of food. Taste buds contain specialized taste receptor cells for detection of different taste qualities. Sweet taste perception is mediated by the lingual T1R3 and T1R2 taste receptors (8). These receptors relay to the primary taste cortex, which is suggested to be located in the frontal operculum and insula in humans. The primary taste cortex is involved in the identification of taste and the perception of taste intensity (9). It is proposed that the hedonic evaluation of taste stimuli involves a secondary, reward-related taste cortex comprising orbitofrontal cortex, anterior cingulate, amygdala, and prefrontal cortices (10, 11): fMRI studies indicate that activation in these areas correlates with the subjective pleasantness of taste (12, 13).

Although it is commonly understood that consuming foods high in sugars is associated with activation of the mesolimbic (midbrain and striatum) reward areas, in addition to gustatory areas (14, 15), there is a paucity of research on basic neural representations of taste and reward responses to simple caloric sweeteners in humans. Despite this limited empirical base, some authors have argued that there are fundamental differences in the neural representation of reward between caloric and noncaloric sweeteners (16, 17). Specifically, it has been suggested that caloric sweeteners activate the reward system, whereas noncaloric sweeteners do not (16–19).

WHO has produced guidelines for the reduction of sugar intake for adults and children (18). Consequently, many food and drink products that have traditionally had high sugar content are increasingly available as low-calorie versions, formulated with noncaloric sweeteners as alternatives to sugars. Potential differences in reward responses to sugars and noncaloric sweeteners could have important implications for the sustained acceptance of reformulated food products. For example, even when formulated to achieve similar sweetness concentrations and initial acceptance, foods lower in sugars might fail to sustain consumer appeal because they may lack some intrinsic elements that give rise to reward. Unfortunately, our understanding of possible differences in reward processing of sugars and nonnutritive sweeteners is limited by the fact that different fMRI studies report heterogeneous activation foci for sweet tastes and are associated with methodological and analytical inconsistencies [e.g., variable stimulus intensities, different modes of delivery, and whole brain compared with region of interest (ROI) analyses]. Moreover, much of the published neuroimaging research uses small sample sizes (typically *n* <20), thus reducing the reliability of the data (19).

Murray et al. (4) recommend exploration of the comparability of the pleasure and satisfaction derived from consuming original and reformulated, reduced-sugar products. Consensus on the impact of sugars on central mechanisms is required in order to determine the relative impact of noncaloric sweeteners on taste signaling and reward processing. Consequently, it is necessary to constrain analyses to the effects of the taste of sugars, independent of other orosensory factors. This goal may be addressed, despite the limitations of the existing data, through meta-analysis: By pooling data from published work on neurophysiological responses to sweet tastants, we may establish a more consistent picture of regional brain activations associated with gustatory and reward processing.

The meta-analyses reported here aimed to compare brain regions activated in response to tasting caloric sweeteners (the contrasts being brain activity during receipt of sucrose/sugars minus brain activity during receipt of control/tasteless solution) in healthy participants, using the published fMRI data to produce a consistent brain map of activation induced by sweet taste. The primary objective was to clarify the brain regions associated with caloric sweet taste sensation and reward. Understanding how caloric sweet taste is represented in the brain will have important implications for reformulation of high-sugar foods to have reduced energy content.

## Methods

### Data search and extraction

#### Information sources and search strategy

The formal search strategy consisted of systematically examining 3 electronic databases through October 2019 (MEDLINE, Scopus, and PsycINFO) using the MeSH search terms fMRI AND (glucose OR sucrose OR fructose OR maltodextrin OR sucralose OR stevia OR steviol OR glycosides OR aspartame OR saccharin OR saccharine OR sugars OR sweetener). Searches were restricted to terms found in the title or abstract of the articles. No date limit was set for the searches.

Manual searches of the reference sections of identified articles were conducted to supplement the formal searches. Previous meta-analyses of activation likelihood estimation (ALE) on human gustatory cortex and basic taste (20, 21) were also screened for additional articles.

#### Article selection and extraction of data

Formal database searches were conducted by 2 authors independently (CAR and NF), as were supplementary and manual searches. Both authors were responsible for assessment of articles for inclusion, and decisions regarding article inclusion were determined by discussion. One author (CAR) extracted the relevant data, and these were cross-checked by a second author (NF).

#### Eligibility criteria

The criteria for inclusion were *1*) any human fMRI studies published through October 2019; *2*) original English language articles; *3*) published in a peer-reviewed journal; *4*) used pure tastants dissolved in water (not emulsions or milkshakes); *5*) employed a contrast between a sugar/sweetener solution and either a control solution (water or tasteless solution) or baseline activity (whereby activity in the control condition was subtracted from activity in the experimental condition; i.e., activation rather than deactivation); *6*) coordinates were reported in the article or supplementary material in Montreal Neurological Institute [MNI (22)] or Talairach space (23); and *7*) data were obtained from a healthy (including overweight or obese) population (systemic disease-free).

#### Additional handling of data

Some fMRI studies do not analyze the whole brain but rather focus on 1 or more specific, predefined ROIs. We did not exclude articles that reported ROI results, although ROI data may bias ALE meta-analyses (24, 25). Instead, we included ROI data in supplementary analyses with the data from whole brain analyses and those using a large mask covering the gustatory cortex (for these additional analyses, see **Supplemental Tables 1–4**). However, in those instances in which both whole brain and ROI significant coordinates were reported [e.g., Nakamura et al. (26)], only the whole brain coordinates were included in our ALE meta-analysis.

Studies that reported coordinates in the Talariach space (16, 27–33) were converted into MNI coordinates using GingerALE (Brainmap GingerALE version 2.3.6; Research Imaging Institute; http://brainmap.org).

Other decisions about data selection included reporting the coordinates from the fasting condition but not the satiated condition from Haase et al. (30) due to the fasting condition arguably being more likely to report greater general reward activation (34); reporting coordinates from the “non-diet soda drinkers” rather than “diet soda drinkers” in Green and Murphy (28)—that is, a group that had not attained a conditioned taste preference for nonnutritive sweeteners; and reporting coordinated from “young adults” and not “old adults” in Jacobson et al. (31) because taste perception may change with age (35) and due to the other included studies in our analysis being conducted on young adults.

#### Activation likelihood estimation meta-analysis

Two primary ALE meta-analyses were conducted: one for experiments using sucrose as the sweet stimulus (as this was the case in the majority of studies) and a separate analysis for all experiments using any caloric sweeteners (this included the sucrose studies with the addition of studies conducted using glucose). See **Table 1** for data on sweetener concentrations, sweetness, intensity, and pleasantness of taste stimuli. Each of these primary analyses was repeated with the inclusion of ROI studies (Supplemental Tables 1–4). To be included in the sucrose-only meta-analysis, data must have been analyzed with the direct contrast between sucrose and either a control solution (water or tasteless solution) or baseline activity (experimental condition minus control condition activation). For the all-sugars meta-analysis, data had to be derived from a direct contrast between a caloric sweetener (sucrose, glucose, or fructose) and either a control solution or baseline activity (experimental condition minus control condition). For completeness, we also included descriptions of the few available studies on noncaloric sweetener in **Supplemental Table 5**.

In order to determine consistency in reported regions of neural activation for both analyses, we conducted coordinate-based ALE meta-analyses (single data set analysis). The analyses were performed using Brainmap GingerALE version 2.3.6. The algorithms in this software assess the spatial convergence of foci using the reported coordinates of activation peaks from the individual studies (rather than peak height/signal intensity). These algorithms use kernel techniques for assessing spatial uncertainty around the reported peaks (36). The overlap between kernels is calculated to determine spatial location convergence that is greater than that expected by chance. Our meta-analysis is of spatial convergence across studies using the (*x, y, z*) coordinates from individual studies of peak activations.

We adhered to the ALE method (http://www.brainmap.org/ale) of Eickhoff et al. (24, 37) that uses a random effects model to assess agreement across experiments in reported coordinates. We also applied a correction devised by Turkeltaub et al. (25) that minimizes within-experiment effects (differences in number of reported foci that are in close proximity, which affects an individual experiment's contribution to an ALE map) and within-group effects (multiple contributions from the same sample, with the same contrast within the same article). Therefore, an ALE value represents the degree of concordance in activation across independent studies. This method assigns an ALE value to each voxel (1-mm^3^ volumes of brain tissue): ALE values increase with the number of studies that report activated peaks at a voxel or in close proximity. Thus, consistency of voxel activation across studies can be assessed.

Standardized procedures for performing ALE using GingerALE are reported in the GingerALE user manual (Research Imaging Institute, 2013), and recent recommendations on methodology have been reported by Eickhoff et al. (38). In brief, modeled activation (MA) maps were produced for each experiment using reported coordinates in MNI space. In ALE meta-analysis, each set of peak coordinates from an individual study is entered into an empty brain. The voxels within that cluster are given a value of 1, and all other voxels in the brain (∼100,000) are given a value of 0 (37). The MA map consists of each of the reported coordinates from an individual study being entered, and then a smoothing procedure is performed whereby the value of 1 is smeared out to neighboring voxels using a Gaussian kernel. The degree of this smoothing out is based on the sample size. This is because smaller samples have less statistical power and a greater spatial uncertainty; therefore, smaller sample sizes lead to increases in kernel sizes (37).

Each voxel within the map has an MA score that reflects the likelihood of that location having fMRI activation (37). The MA score is based on a 3D normal probability distribution centered on the entered coordinates (21). Following this procedure, the individual MA maps were combined into 1 ALE map that represents the union of probabilities. An ALE value at each voxel (with coordinates *x, y, z*) is calculated by taking the union of probabilities from individual MA maps at that voxel divided by the number (*k*) of studies in the meta-analysis. Using this ALE map, true convergence of activation foci was then distinguished from random clustering (noise) by testing against the null hypothesis (by creating a null distribution map) that there is a random spatial association between experiments (38).

A *P* value was calculated for each voxel based on probabilities of attaining an ALE value that differed from that of the corresponding voxel on a null-distribution map, via random permutation. We used the same number of threshold permutations as those reported in Yeung et al. (21). Thus, the *P* values in our analyses were generated by 5000 permutations (39, 40).

In all analyses, we adhered to the recommendations of Eickhoff et al. (38) by using a cluster-level family-wise error (few) at *P* < 0.05 to correct for multiple comparisons, following an initial cluster forming threshold of uncorrected *P* < 0.001. Relative to voxel-level FWE, cluster-level FWE is suggested to be more sensitive due to its superior power to voxel inference (41, 38) while still controlling for incidental convergence. Cluster-level FWE thresholding provides an appropriate compromise between sensitivity and specificity. Multi-image analysis GUI (http://ric.uthscsa.edu/mango) was used to overlay ALE maps onto an anatomical image using MNI coordinates.

## Results

**FIGURE 1:**
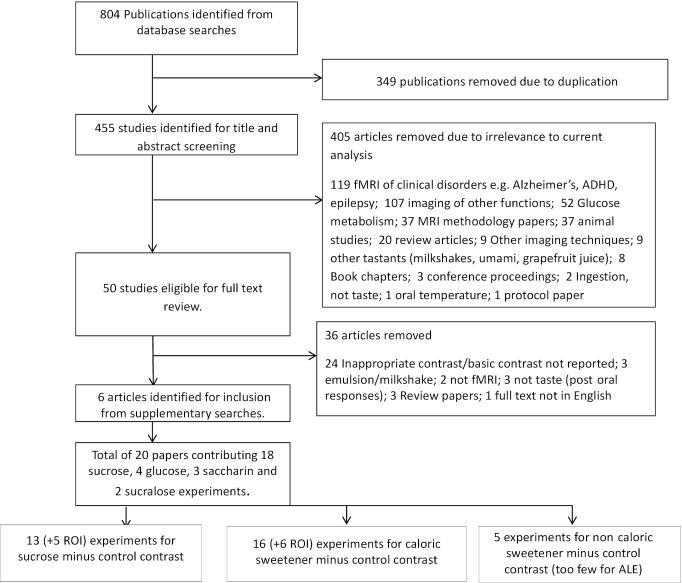
Flow diagram of study selection from systematic review of fMRI studies assessing sweet taste in human adults. ADHD, attention deficit hyperactivity disorder; ALE, activation likelihood estimation; ROI, region of interest.

**Figure 1** shows a flow diagram indicating the study selection steps. A total of 804 articles were returned from the initial searches (PsycInfo, 151; MEDLINE, 239; Scopus, 414). Of these, 349 were duplicates and were removed in the first step. A further 405 articles were removed following the initial review of titles and abstracts. Studies excluded at this stage included those examining clinical populations (119), those using fMRI to examine other functions (107), glucose metabolism studies (52), MRI methodology articles (37), animal studies (37), review articles (20), studies employing non-fMRI techniques (9), studies employing other tastants (milkshakes, umami, grapefruit juice) (9), book chapters (8), conference proceedings (3), studies investigating ingestion rather than taste (2), a study investigating oral temperature manipulation (1), and 1 protocol article. A further 36 studies were removed following full-text review (for details, see Figure 1). An additional 6 studies were identified via supplementary searches, leaving a total of 20 studies that met our eligibility criteria. Five of these conducted ROI analysis only and so are restricted to supplementary analysis (Supplemental Tables 1–5). A total of 15 studies contributed to the 2 primary analyses.

The final sample of studies in our analysis includes healthy weight, overweight, and obese participants, although as can be seen from Table 1, samples are primarily composed of participants with a BMI (in kg/m^2^) <25. Studies that focused on activation in response to milkshakes or emulsions were excluded from the reported analysis. Inclusion of such studies may yield different results compared with those reported here on pure tastants. For more detailed summary data, see Table 1.

**FIGURE 2:**
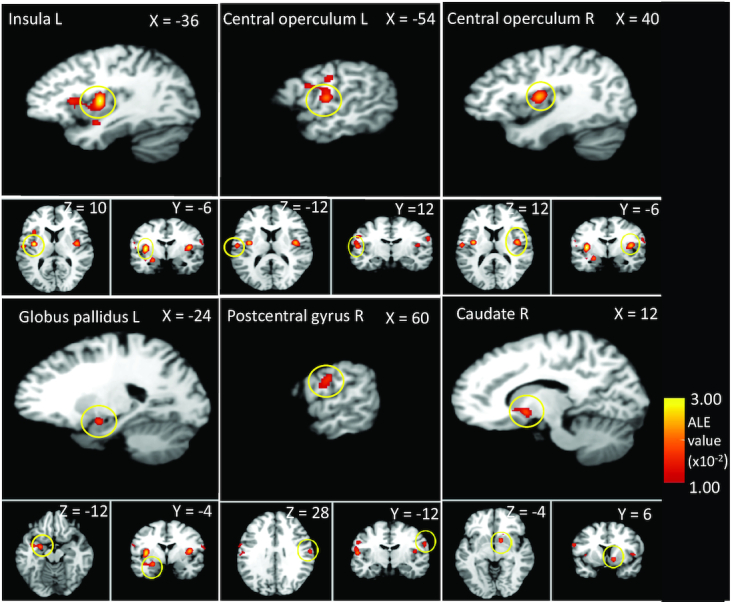
Localization of significant ALE clusters from the sucrose minus control contrast, from ALE meta-analysis of sweet taste in human adults. GingerALE output overlaid onto a standard template (Colin27_T1_seg_MNI.nii) in MNI space. ALE, activation likelihood estimation; MNI, Montreal Neurological Institute.

**FIGURE 3:**
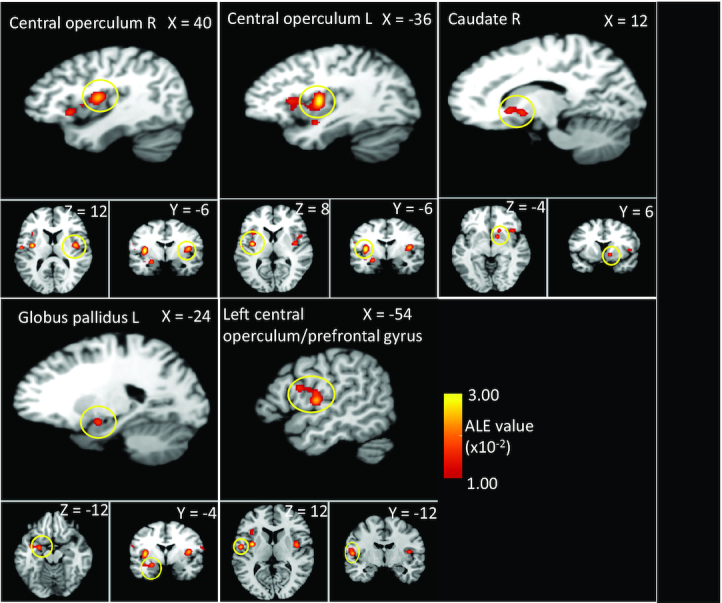
Localization of significant ALE clusters from the sucrose minus control and glucose minus control contrast studies combined meta-analysis of sweet taste in human adults. GingerALE output overlaid onto a standard template (Colin27_T1_seg_MNI.nii) in MNI space. ALE, activation likelihood estimation; MNI, Montreal Neurological Institute.

**TABLE 1:** Studies and experiments included in ALE meta-analyses on sweet taste in human adults^1^

Study and year	Contrast	Concentration	Pleasantness/intensity	Hunger state	*n* (men)	Age (y)	Hand	BMI	Foci/clusters	Statistical correction	Whole brain/mask
Sucrose whole brain experiments											
Small et al. (42) 2003	Sucrose minus tasteless solution	Two concentrations tailored to each participant (range: 0.018–0.056 M), matched by pleasantness and intensity	Stimuli rated as weak pleasant or intense pleasant	—	9 (3)	24	R	—	14: Putamen, claustrum, hypothalamus, caudolateral OFC, anterior ventral insula, subcallosal cingulate, anterior cingulate	FWE	Whole brain
Haase et al. (29) 2007	Sucrose minus water	0.64 M	Stimuli evaluated as pleasant	—	18 (9)	20.7 ± 1.0	—	—	14: Postcentral gyrus, precentral gyrus, dorsal anterior insula, dorsal posterior insula, rolandic operculum, BA 13, IFG, thalamus, caudate	Monte Carlo	Whole brain
Kami et al. (43) 2008a	Sucrose minus water	0.5 M	Moderate to strong sweetness Pleasantness not assessed	—	3 (0)	36.3 ± 6.8	—	—	1: Insula	Uncorrected *P* < 0.001	Whole brain
Kami et al. (43) 2008 b	Sucrose minus water	0.5 M	Moderate to strong sweetness Pleasantness not assessed	—	3 (0)	36.3 ± 6.8	—	—	1: Insula	Uncorrected *P* < 0.001	Whole brain
Frank et al. (16) 2008	Sucrose main effect	0.29 M	Pleasantness slopes were calculated that predicted left insula activity	Sated following breakfast	12 (0)	27 ± 6	—	22 ± 2	10: Frontal operculum/insula/claustrum, midsagittal cingulate gyrus, superior frontal gyrus, anteroventral striatum with nucleus accumbens, thalamus, midbrain (substantia nigra, ventral tegmental area), temporal operculum, dorsal caudate	Main effect statistical maps were thresholded at *P* < 0.005 (minimum of 16 contiguous voxels)	Whole brain
Green and Murphy (28) 2012	Sucrose minus water	0.64 M	Mean pleasantness rating: 54 ± 14.3; mean intensity rating: 33.3 ± 12.3 (out of 100)	Fasted for 12 h	12 (5)	23.0 ± 2.3	—	25.03 ± 5.6	15: Precentral gyrus, postcentral gyrus, precuneus, cerebellum, thalamus, cingulate gyrus, inferior parietal lobule, paracentral lobule, insula, superior parietal lobule	Monte Carlo	Whole brain
Haase et al. (30) 2009	Sucrose minus water	0.64 M	Pleasantness not discussed	Fasted for 12 h	18 (9)	20.7 ± 1.0	—	Men = 24.4; women = 23.03	38: Insula, BA 13, frontal operculum, OFC, amygdala, para hippocampus, middle frontal gyrus, claustrum, thalamus, lentiform nucleus, hypothalamus, caudate, substantia nigra, cerebellum, cuneus, culmen, cingulate gyrus, postcentral gyrus, rolandic operculum, superior temporal gyrus, medial frontal gyrus, anterior cingulate, precuneus, fusiform gyrus, caudate body	Monte Carlo	Whole brain
Jacobson et al. (31) 2010	Sucrose minus baseline	0.64 M	No data on taste or intensity presented	Fasted for 12 h	19 (10)	23.9	—	—	17: Insula, precentral gyrus, rolandic perculum/BA 43, postcentral gyrus, transverse temporal gyrus, claustrum, cerebellum, inferior frontal gyrus, middle temporal gyrus, anterior cingulate, cerebellum, parahippocampal gyrus	Monte Carlo	Whole brain
Eldeghaidy et al. (44) 2011	Sucrose minus tasteless solution	0.09 M	—	Tested 2 h after a light breakfast	13 (6)	28 ± 8	R	24 ± 4	19: Thalamus, SI, SII, rolandic operculum, frontal operculum, anterior insula, mid-insula, insula/claustrum, putamen, rostral anterior cingulate cortex, anterior cingulate cortex, posterior cingulate, amygdala, piriform, medial OFC, inferior frontal gyrus, medial frontal gyrus, mid-frontal gyrus, superior frontal gyrus, precentral gyrus, superior temporal gyrus, precentral gyrus, superior temporal gyrus, fusiform, supramarginal gyrus/inferior parietal lobule, caudomedial OFC	Uncorrected *P* < 0.01 + *k* > 5	Whole brain
Nakamura et al. (26) 2012	Sucrose minus tasteless solution	0.5 M	Sweet taste intensity rated at 7.1 ± 0.3 (out of 10) Pleasantness not rated	Fasted for 2 h	20 (10)	24.2 ± 2.7	—	—	1: Left middle insula cortex	FWE	Whole brain
Kareken et al. (45) 2013	Sucrose minus water	0.83 M	Perceived intensity: 62.0 ± 8.8; perceived pleasantness: 59.6 ± 18.5 (out of 100)	Participants asked to eat usual breakfast 1 h before arrival	16 (12)	—	R	25.9 ± 3.2	22: Posterior, dorsal insula/frontal operculum, frontal opercular cortex, inferior frontal gyrus, middle frontal gyrus, dorsal amygdala, middle frontal gyrus, ventral striatum/nucleus accumbens, precentral gyrus, OFC (anterior orbital gyrus), parietal opercular cortex, postcentral gyrus, ventral anterior insula, postcentral gyrus, uncus/amygdala, middle frontal gyrus, inferior frontal gyrus, middle cingulate, ventral insula, posterior orbital cortex (medial orbital gyrus), supplementary motor area, ventral postcentral gyrus	Uncorrected *P* < 0.001	Whole brain
Avery et al. (27) 2015	Sucrose minus tasteless solution	0.6 M	Intensity rated at 6.1 ± 2.5; sweetness rated as 7.5 ± 1.5; pleasantness rated as 6.1 ± 2.9 (out of 10)	—	20 (12)	28 ± 7	R	29 ± 6; range: 20–43	7: Postcentral gyrus, precentral gyrus, dorsal mid-insula, inferior frontal gyrus	Monte Carlo	Whole brain
Eiler et al. (46) 2018	Sucrose minus tasteless solution	0.83 M and 0.10 M (activation for both sucrose solutions)	0.83 M rated as more intense than 0.10 M sucrose solutions	Fed a standardized breakfast 3 h before testing	74 (32)	—	R	—	20: Posterior insula, ventral insula, anterior insula, OFC, postcentral gyrus, precentral gyrus, middle frontal gyrus, dorsal amygdala, supramarginal gyrus, ventral striatum	FWE	Whole brain
Total	13 experiments				241				179		
Experiments with sugars other than sucrose											
Chambers et al. (47) 2009	Glucose minus tasteless solution	0.5 M	Sweetness = 55 ± 21; pleasantness = 64 ± 8 (out of 100)	Overnight fast	7 (7)	23 ± 3	R	22.2 ± 1	7: Insula/operculum, DLPFC, striatum, cingulate cortex	One-tailed *P* = 0.003	Whole brain
Chambers et al. (47) 2009	Glucose minus control	1.0 M		Overnight fast	7 (5)	24 ± 2	R	22.7 ± 0.7	9: Insula/operculum, OFC, DLPFC, striatum, cingulate cortex	—	Whole brain
O'Doherty et al. (48) 2001	Glucose minus tasteless solution	1.0 M	Pleasantness (from 2 very pleasant, 0 neutral, –2 very unpleasant) = 0.9 ± 0.49	—	7 (—)	—	—	—	14: OFC, insula/operculum, anterior cingulate, anterior temporal lobe, inferior prefrontal cortex, premotor, striatum	Uncorrected *P* < 0.01 + *k* > 3	Whole brain
Totals	3 experiments				21				30		

1 Values are means ± SDs or ranges unless otherwise indicated. BA, Broca's area; DLPFC, dorsolateral prefrontal cortex; FWE, family-wise error; IFG, inferior frontal gyrus, *k*, cluster size in units of contiguous clusters; MNI, Montreal Neuroimaging Institute; NA, not applicable; OFC, orbitofrontal cortex; R, right; ROI, region of interest; SI, somatosensory cortex; SII, secondary somatosensory cortex.

### Significant ALE clusters for the sucrose minus control contrast

The sucrose minus control contrast ALE meta-analysis pooled the data from 13 eligible experiments (from 12 articles, with a total of 241 participants and 179 reported foci). The results (**Table 2**, **Figure 2**) revealed 6 significant clusters. The largest of these involved the left insula and frontal operculum; however, we also saw activations in bilateral central operculum, postcentral gyus, left precentral gyrus and globus pallidus, and right frontal operculum and caudate (**Supplemental Data Files 1** and **2**).

### Sensitivity analysis

To address small study bias in our main analysis, we repeated the sucrose minus control ALE analysis following the removal of studies with an *n* <10 (**Supplemental Data File 3**). This revealed stability of the 6 clusters produced in the primary analysis and showed an additional cluster in the right insula (primary taste area). See Table 2 for comparison of primary and sensitivity analyses.

A further sensitivity analysis was conducted to observe stability of results following the removal of a single dominant study with a large sample size (**Supplemental Data File 4**). This was conducted due to Eiler et al. (46) (*N* = 74) contributing ∼30% of the total sample size in the primary meta-analysis. After removal of data from Eiler et al. (46), the right caudate and bilateral postcentral gyrus clusters were no longer significant (Table 2).

These sensitivity analyses were supplemented by an additional 12 “leave one out” analyses, whereby the primary analysis was rerun, each time excluding a different single study. These analyses did not contribute any additional information to the reported sensitivity analysis.

### Significant ALE clusters for the sucrose minus control and glucose minus control contrast studies combined

In addition to the sucrose-only analysis (sucrose minus control contrast), we added the experiments that provided coordinates for glucose minus control contrasts. Included in this analysis were 15 studies (contributing 16 experiments) with a total of 262 participants and 209 reported foci. The analysis pooled data from the 13 eligible experiments analyzed in the sucrose minus control contrast, plus a further 3 experiments using glucose minus control contrasts (47, 48) (**Supplemental Data File 5**). Together, the ALE from these 16 experiments produced 5 significant clusters (**Table 3**, **Figure 3**, **Supplemental Data File 6**). These clusters related to right central operculum, anterior insula and frontal operculum, left central operculum and anterior insula, right caudate, left globus pallidus and mid-insula, and left central operculum and precentral gyrus.

### Sensitivity analysis

The 3 glucose minus control contrast studies had small samples (*n* <10). Thus, the results of an analysis after removal of small sample studies are the same as those reported in the sucrose sensitivity analysis for small sample bias. However, following removal of Eiler et al. (46), there were 4 significant clusters. Importantly, caudate and left globus pallidus were no longer significant clusters (Table 3, **Supplemental Data File 7**).

**TABLE 2:** Locations (MNI) of significant clusters from the contrast sucrose minus control from an ALE meta-analysis of sweet taste in human adults^1^

		Contributing experiments		Peak voxel coordinates^2^			
Cluster size (mm^3^)	Brain region	*n* (refs)	%	*x*	*y*	*z*	ALE value
Primary analysis^3^							
3008	Insula L	9 (16, 26, 27, 29–31, 44–46)	69.2	−36	−6	10	0.0299
	Insula L			−32	16	2	0.0174
	Frontal operculum L			−34	18	8	0.0163
2736	Central operculum L	7 (27, 29–31, 44–46)	53.9	−54	−12	12	0.0212
	Precentral gyrus L			−58	2	24	0.0198
	Postcentral gyrus L			−52	−14	32	0.0145
	Postcentral gyrus L			−60	−16	24	0.0134
2368	Central operculum R	10 (16, 27–30, 43–46)	76.9	40	−6	12	0.0249
	Frontal operculum R			46	10	4	0.0154
1240	Globus pallidus L	4 (1, 30, 45, 46)	30.8	−24	−4	−12	0.0199
	Insula L			−38	−2	−12	0.0148
848	Postcentral gyrus R	4 (28, 29, 44, 46)	30.8	60	−12	28	0.0161
768	Caudate R	4 (29, 30, 45, 46)	30.8	12	6	−4	0.0183
	Caudate R			16	16	0	0.0121
Sensitivity analysis with reference (46) removed							
2472	Mid-insula L/	8 (16, 26, 27, 29–31, 44, 45)	66.7	−34	−6	12	0.0257
	Frontal operculum L			−36	20	10	0.0142
2312	Central operculum L/	6 (27, 29–31, 44, 45)	50.0	−54	−12	12	0.0212
	Precentral gyrus L			−58	2	24	0.0198
1400	Central operculum R	7 (27–30, 43, 45)	58.3	42	−8	14	0.0205
728	Frontal operculum R	3 (16, 30, 44)	25.0	46	10	4	0.0154
Sensitivity analysis with references (42) and (43) removed							
2896	Central operculum L	7 (27, 29–31, 44–46)	70	−54	−12	12	0.0212
	Precentral gyrus L			−58	2	24	0.0198
	Postcentral gyrus L			−52	−14	32	0.0145
	Postcentral gyrus L			−60	−16	24	0.0136
2800	Insula L	9 (16, 26, 27, 29–31, 44–46)	90	−36	−6	10	0.0299
	Insula L			−32	16	4	0.0163
	Frontal operculum L			−34	18	8	0.0162
1312	Globus pallidus L	4 (30, 44–46)	40	−24	−4	−12	0.0198
	Insula L			−38	−2	−12	0.0146
1240	Central operculum R	6 (27–30, 45, 46)	60	40	−6	14	0.0211
896	Postcentral gyrus R	4 (28, 29, 44, 46)	40	60	−12	28	0.0161
824	Caudate R	4 (29, 30, 45, 46)	40	12	6	−4	0.0183
	Caudate R			14	16	0	0.0121
592	Insula R	3 (30, 45, 46)	30	40	6	−12	0.0186

1 All *P* values < 0.001. ALE, activation likelihood estimation; L, left; MNI, Montreal Neuroimaging Institute; R, right.

2 Using the anterior commissure as the origin of the MNI coordinate system: *x* = from left to right, *y* = from posterior to anterior, and *z* = from inferior to superior.

3 Total number of experiments for primary analysis = 13.

## Discussion

The current meta-analyses found that the brain regions most consistently activated in response to tasting caloric sweeteners were in the primary taste areas of the mid-insula, anterior insula, frontal operculum, central operculum, precentral gyri, and thalamus. The mid-insula is frequently cited as being a primary region of the human taste cortex (9, 49, 50). The anterior insula is also a primary cortical taste region that integrates information about different tastes, as well as information about the texture and temperature of oral stimuli (9). Similarly, the operculum is an important region for conscious taste perception that has projections from the tongue in primates (51, 52), and its activation has been reported in previous fMRI meta-analyses of basic taste responses (20, 21), as well as in other neuroimaging modalities [e.g., gustatory evoked potentials (53)]. The precentral gyrus is often argued to be an important area of the brain for taste projection: Lesions in this region are associated with taste deficits, both in humans and in primates (54), and surgical ablation of this area can suppress gustatory hallucinations in epileptics (55). The thalamus is also integral to processing of taste intensity, with an fMRI study suggesting that varying (salt) taste intensity modulates effective connectivity from the insula to the thalamus in humans (56).

In each of our main analyses we observed activity in the caudate, a structure of the dorsal striatum that has a role in reward/salience orientation (47, 57). Although signifying reward responses to sugars, it is important to note that this activation derives predominantly from the data of a single study. The caudate cluster was the smallest and third smallest cluster, respectively, in our 2 primary analyses. Crucially, activation of the caudate was no longer evident in our sensitivity analyses following the exclusion of the most dominant, large-sample study of Eiler et al. (46). Furthermore, in none of our analyses was there evidence of consistent orbitofrontal cortex activity, an area of the secondary taste cortex suggested to be involved in reward processing (9, 10, 58).

**TABLE 3:** Locations (MNI) of significant clusters from the contrasts sucrose minus control and glucose minus control from an ALE meta-analysis of sweet taste in human adults^1^

		Contributing experiments		Peak voxel coordinates^2^			
Cluster size (mm^3^)	Brain region	*n* (refs)	%	*x*	*y*	*z*	ALE value
Primary analysis^3^							
3456	Central operculum R	12 (16, 27–31, 43–46, 48)	75	40	−6	12	0.0250
	Anterior insula R			38	18	2	0.0187
	Frontal operculum R			46	10	6	0.0163
3424	Central operculum L	11 (16, 26, 27, 29–31, 42, 44–46, 48)	68.8	−36	−6	8	0.0300
	Anterior insula L			−30	16	4	0.0202
1136	Caudate R	5 (29, 30, 45–47)	31.3	12	6	−4	0.0184
	Caudate R			14	18	−2	0.0171
1120	Globus pallidus L	4 (30, 44–46)	25	−24	−4	−12	0.0199
	Mid-insula L			−38	−2	12	0.0149
896	Central operculum L	4 (29, 30, 44, 45)	25	−54	−12	12	0.0212
	Precentral gyrus L			−56	4	24	0.0190
Sensitivity analysis with reference (41) removed							
2856	Mid-insula L	8 (16, 26, 27, 29–31, 44, 45)	53.3	−34	−6	12	0.0258
	Mid-insula L			−40	0	2	0.0148
	Frontal Operculum L			−36	18	10	0.0143
	Anterior insula L			−32	16	0	0.0121
2224	Central operculum L	6 (27, 29–31, 44, 45)	40.0	−54	−12	12	0.0212
	Precentral gyrus L			−58	2	24	0.0202
1336	Frontal operculum R	4 (16, 30, 31, 44)	26.7	46	10	6	0.0163
	Insula R			30	16	2	0.0116
1312	Central operculum R	7 (27–30, 43, 45)	46.7	40	−6	14	0.0206

1 All *P* values < 0.001. ALE, activation likelihood estimation; L, left; MNI, Montreal Neuroimaging Institute; R, right.

2 Using the anterior commissure as the origin of the MNI coordinate system: *x* = from left to right, *y* = from posterior to anterior, and *z* = from inferior to superior.

3 Total number of experiments for primary analysis = 16.

It is apparent, therefore, that the data from the majority of studies analyzed provide limited evidence of sweet taste-induced reward-related activation, and observation of the expected regional reward activation in our meta-analyses depended on the inclusion of a single, large sample study (46). Consequently, it may be premature to draw strong conclusions regarding a hedonic brain response to caloric sweetness within the existing database. This weakness reflects the predominance of underpowered studies in this field, and it demonstrates that more large-sample experiments are essential if we are to further our understanding of sweet taste-reward processing.

Although several articles have reported increased mesolimbic reward area activation to caloric sweet taste (e.g., 16, 17, 30, 46, 47), it may not be surprising that our sensitivity analysis de-emphasized these areas. There could be several reasons for this; as previously indicated, our findings reflect the predominantly small sample sizes of the individual experiments included in the ALE. However, there are also several sources of heterogeneity across the studies included—for example, variation in the data analytic methodologies adopted by the original studies. Furthermore, there are specific limitations on our interpretation that derive from the heterogeneous design of the different experiments. For example, there is considerable heterogeneity in the concentration of sucrose stimuli administered across studies, ranging from sapid to insipid. In addition, critical to any examination of reward-related activation, subjective ratings of the pleasantness of test solutions that would establish their rewarding nature are often not reported or measured. Similarly, the specific mode of stimulus delivery varies across experiments—and is not described in some reports. The populations across the included studies were also heterogeneous—for example, European (59), American (29), and Asian (28). It is possible that neural responses to sugars may be heterogeneous across regional and ethnic populations. It may be appropriate to describe the results of our analysis as reflecting the lack of consistency and large heterogeneity in patterns of reward response across studies.

The mode of administration of taste stimuli in the studies that we assessed (**Table 4**) may be particularly critical in explaining inconsistent reward responses. For example, delivery of small volumes of taste solutions into the mouth while participants are lying down, along with specific measures to avoid muscular artifacts from tongue movement or swallowing, may restrict the ecological validity of many experiments. Direct, carefully controlled application of small volumes of sweet solutions to the tongue may inadequately model the usual oral experience of tasting and hedonic evaluation. Such experimental controls could unintentionally interfere with the full complement of behavioral components that may be necessary for the normal experience of pleasure and, consequently, the ability to detect activation of brain reward regions. Perhaps reward-related activations would be more reliably obtained with sweet tastes that are presented in a vehicle that better simulates the more usual properties of ingesta and experience of tasting. Indeed, reward area activation has been detected in studies with sweet milkshakes containing both fat and sugar, with the fat providing texture and mouthfeel that might support hedonic evaluation (15, 60). In addition, similar elicited brain responses were detected for both sugar- and artificially sweetened yogurt drinks (61). The neural response to fat and sugar mixes is therefore a recommendation for future meta-analyses.

Findings from studies with more complex stimuli perhaps reflect the importance of orosensory stimuli beyond mere taste in the activation of reward systems. Although many taste neurons have been identified in the orbitofrontal cortex of primates (52), there are also other aspects of orosensory experience represented there, such as texture (62) and olfactory stimuli (63). Possibly, activation of a combination of these sensory aspects is required to produce the full neural representation of pleasure, with the processing of multiple sensory inputs and supra-additive activity in specific brain regions being necessary to experience the pleasantness of food (64).

**TABLE 4:** Summary of mode of delivery of oral solutions from studies included in the ALE meta-analyses of sweet taste in human adults^1^

Study	Mode of delivery
Small et al. (42)	Solution delivered using a calibrated dropping pipette at 0.5 mL during a 5-s period. Participants cued to swallow 15 s after onset of solution delivery in an event-related design. Each trial was followed by a 5-s rinse. Data were analyzed from 5 to 15 s of the trial.
Kami et al. (43)	A computerized system controlled timing and duration of solution delivery (8 s of stimulus delivery followed by 16 s of deionized water per trial, in a block design) using solenoid valves. Solutions were delivered into an intra-oral device in which the participants placed the tip of their tongue (solutions did not leak into the whole mouth). The device was held in place by an individual rigid mouthpiece.
Hasse et al. (29)Hasse et al. (30)Green and Murphy (28)Jacobson et al. (31)	Solution delivered to the tip of the tongue through tubes encased in a dental wax-covered bite bar. Solutions delivered at 0.3 mL/s via syringes connected to programmable pumps. Pleasantness and intensity were rated in the scanner using a joystick. Solutions delivered for 1 s followed by 2 s cued swallow, 1 s presentation of psychophysical rating instructions (pleasantness and intensity), and 6 s for rating intensity and pleasantness. Two water rinses followed each trial, in an event-related design.
Frank et al. (16)Oberndorfer et al. (33)	1-mL fluid samples were delivered through ¾-in. tubing into the middle of the mouth via a semi-automatic programmable customized syringe pump. Trials were separated by 20 s.
Eldeghaidy et al. (44)Kareken et al. (45)	Solutions were administered using an automatic spray delivery system, whereby stimuli were gently sprayed across the oral cavity to give extensive coverage of the tongue and mouth before swallowing. 3 mL of solution was sprayed for 3 s (1 mL/s); 18 s later, 2 rinses of control solution were delivered for 5 s each. Following the second control solution rinse, the cycle was repeated. Participants cued to swallow immediately upon cessation of stimulus delivery.
Nakamura et al. (26)	Solution delivery system connected to a suction apparatus that negated the need to swallow. Flow rate kept stable at 1.83 mL/s using flowmeters. The trial proceeded with 15 s of tasteless solution followed by 6 s of stimulus solution.
Avery et al. (27)	0.4 mL of solution delivered onto tongue for 5 s, followed by a 2.5- to 12.5-s delay before a rinse and swallow with distilled water.
De Araujo et al. (65)Chambers et al. (47)	Stimuli delivered into mouth through polythene tubes held in lips. 0.75 mL delivered manually under computer instruction. Participants instructed to swallow 10 s after stimulus delivery; after a 3-s delay, participants rated the taste of stimuli. Five seconds after rating, tasteless solution was administered in the same way as the test stimulus. Participants were instructed to swallow after 10 s.
Rudenga and Small (66)Rudenga and Small (11)	Participants received solutions through a gustometer, with each taste delivered at a volume of 1-mL bolus over 3 s. Following stimulus delivery, a rest period of 13–17 s was observed, followed by a cue to swallow, a rinse (1 mL tasteless solution for 3 s), and a second rest and swallow cue. Control condition followed the same procedure.
Monteleone et al. (32)	Tastants delivered via tubes fixed on the lips. Tastes delivered at 1 mL/s. Participants instructed to swish and swallow after delivery and then again after 20-s rinse. Patients then swished and swallowed again, followed by a 10-s rest before the next trial.
O'Doherty et al. (48)	Stimuli delivered intra-orally via polythene tubing. 0.5 mL of taste delivered at the start of an 8-s ON period, followed by 0.5 mL of tasteless solution at the start of an 8-s OFF period. Blocked design.
Eiler et al. (46)	Stimuli delivered intra-orally using a computer controlled gustometer with a spray nozzle that lightly covered the tongue of participants with either 0.75 mL of a sucrose solution (0.83 or 0.10 M) or control stimulus consisting of water + thickening agent. Participants were given the signal “ready” to prepare for the delivery of a solution, followed by “spray.” Participants then held solution in mouth until prompted to swallow (jittered 1–3 s after sprays).

1 ALE, activation likelihood estimation.

### Strengths and limitations of this review

A strength of the current analysis was the stringent, well-defined, and transparent inclusion and exclusion criteria, which enabled an unbiased assessment of the effects of caloric sweeteners based on the totality of directly relevant evidence. However, the literature provided only 5 studies reporting a contrast between a noncaloric sweetener and control solution, so it was not possible to conduct ALE for responses to noncaloric stimuli, alone or in comparison against activation by sugars.

Our sensitivity analysis suggests that reward activity in response to the taste of sugars may be reliably revealed only by having much larger data sets than those presented in the majority of reported studies. Similarly, the putative notion of distinctions between the capacity of caloric and noncaloric sweeteners to activate reward regions may be clarified with more, higher powered experiments. Certainly, given the outcome of our sensitivity analyses in relation to sugar experiments, and the restricted number of studies, any definitive consensus on fundamental differences in reward processing between caloric and noncaloric sweeteners is premature, if not unwarranted by the current evidence (available data on noncaloric sweeteners are summarized in Supplemental Table 5).

Another limitation of meta-analysis is the role of publication bias. Excess reporting of positive findings is known bias in the neuroimaging literature (59). This has several potential sources, such as underreporting of null results, manipulation of thresholds to be more lenient, and ROI analyses. The ROI approach in particular can add publication bias because it allows maximization of finding differences by focusing on brain regions selected a priori (when a strong hypothesis is present) (67). Focusing on a smaller area reduces the number of voxels that are corrected for and thus improves/inflates the possibility of finding a significant result. The caveat to this is that ROI analyses reduce the chance of producing type II error associated with whole brain voxel correction methods. For these reasons, ROI studies are a known bias for ALE meta-analyses techniques, and as such we have sought to reduce this type of bias from our analysis by including only whole brain studies (see **Supplemental Results** for analysis including ROI studies). Nevertheless, there remains the possibility that there are studies that have never been published which could have influenced the findings of the current analysis.

In addition, there are fundamental differences between coordinate-based meta-analytic techniques (e.g., ALE) and traditional effect size-based meta-analytic techniques, namely that coordinate-based meta-analytic techniques use reported coordinates of activation peaks from individual studies. In this way, ALE provides a measure of activation location consistency. Although this is the most appropriate meta-analytic technique for fMRI data (68), it does not provide an effect size of the activations, which would give a usual indication of clinical relevance. For the same reason, this method also does not allow for funnel plots, which provide a method for eyeballing publication bias in a typical meta-analysis.

Finally, a problem for all experiments in this area is the assumed necessity to control for motor responses that might be engendered by the delivery of the tastants. It has been suggested that areas of the insula, thalamus, and precentral gyrus are activated in response to movement (21), which may partly explain some of the activation in these areas detected in the current analyses.

Methodological inconsistencies, including potentially inappropriate choice of stimuli and the absence of definitive subjective measures of pleasantness, represent real obstacles to interpretation of reward-related effects of sweet taste. Another factor may be that sugar stimuli experienced in a scanner, using typically constrained modes of delivery, do not provide a sufficient proxy for normal consummatory experience. It could be that it is the ingestive experience as a whole—combining multiple sensory and behavioral factors—that gives rise to orosensory pleasure, rather than the mere perception of a single taste constituent.

## Conclusion

Using ALE, we have mapped cerebral activations in response to the taste of caloric sweeteners (sucrose and glucose), which primarily recruit the insula, operculum, pre- and postcentral gyrus, and thalamus. We have confirmed the importance of these brain regions for gustatory processing and have provided coordinates in MNI space that may be used for comparison in much needed future studies to define neural responses to sugars and, particularly, noncaloric sweeteners. In accord with previous gustatory ALE analyses, we also recognize the recruitment of a wide cortical network for the processing of sweet taste. Our data should aid the future construction of the necessary consensus on the relative impact of sugars and sweeteners on central taste and reward mechanisms (4) and also support the determination of how behavioral responses to caloric and perhaps noncaloric sweet tastes are underpinned by gustatory-reward network connectivity.

We also report tentative evidence for reward activity in relation to the sweet taste of sugars, with caudate activity being present in our main analyses, but with the caveat that this activity was not confirmed with sensitivity analysis, indicating that reliable effects may be obtained only by having larger data sets such as that reported by Eiler et al. (46). In this light, and with regard to the very limited body of fMRI data, we conclude that it is imperative that more rigorous, higher powered studies be conducted to confirm activation of brain reward regions by sugars.

## Supplementary Material

nxaa071_Supplemental_Files

## Notes

This work was funded by Unilever.

Data described in the manuscript, code book, and analytic code have been submitted as supplementary material and will be made publicly and freely available without restriction.

Registry and registry number for systematic reviews or meta-analysis: As discussed with the editors, this work does not have an associated preregistration.

Author disclosures: CAR, NF, and TCK received grants from Unilever during the conduct of this study. TG, AT, and DJM were employees of Unilever during the conduct of this study. Unilever manufactures food and beverage products containing caloric and noncaloric sweeteners.

Supplemental Tables 1–5 are available from the “Supplementary data” link in the online posting of the article and from the same link in the online table of contents at https://academic.oup.com/jn.

DJM is retired.

Abbreviations used: ALE, activation likelihood estimation; FWE, family-wise error; MA, modeled activation; MNI, Montreal Neurological Institute; ROI, region of interest.
