# Supplementary material for: A Systematic Review and Activation Likelihood Estimation Meta-Analysis of fMRI Studies on Sweet Taste in Humans
Source: J Nutr. 2020 Apr 9;150(6):1619–30. doi: 10.1093/jn/nxaa071 (PMC7269728; doi:10.1093/jn/nxaa071)
Supplement: nxaa071_Supplemental_Files [file nxaa071_supplemental_files.zip › Supplementary results.docx]

***A systematic review and ALE meta-analysis of fMRI studies on sweet taste in humans (Roberts et al.) Online Supplementary Results. Supplementary analyses with inclusion of Region of Interest (ROI) studies.***

*Significant ALE clusters for the sucrose minus control contrast, including ROI studies.*

A further ALE meta-analysis was run to include studies which reported coordinates from ROI analysis or from a large mask covering the gustatory cortex. Data from 17 studies (contributing 18 experiments) were included, with a total of 342 participants and 220 reported foci. This analysis revealed 10 significant clusters in left mid insula and frontal operculum, right frontal and central operculum, left central operculum and precentral gyrus, left central operculum and globus pallidus, right precentral gyrus, right anterior insula, bilateral thalamus, right central operculum and right caudate (see Supplementary Table 1).

*Sensitivity analysis:*

After removal of Eiler et al. (1) there were only 7 significant clusters. Caudate, and left globus pallidus were not named clusters in this analysis (see Supplementary Table 2).

Supplementary Table 1. Locations (MNI) of significant clusters from the contrast sucrose minus control (including ROI experiments), from an ALE meta-analysis of sweet taste in human adults^1^

| Cluster size (mm^3^) | Brain Region | Peak voxel coordinates | | | ALE value | No of contributing experiments | |
| --- | --- | --- | --- | --- | --- | --- | --- |
|  |  | x | y | z |  | *n* | % |
| 4792 | Mid insula L  Frontal operculum L | -36  -36 | -4  14 | 12  8 | 0.0357  0.0282 | 13 | 72.2 |
| 3344 | Central Operculum R  Frontal operculum R | 40  48 | -6  10 | 12  6 | 0.0295  0.0163 | 13 | 72.2 |
|  |  |  |  |  |  |  |  |
| 2696 | Central operculum L  Precentral gyrus L | -54  -58 | -12  2 | 12  24 | 0.0226  0.0198 | 9 | 50 |
|  |  |  |  |  |  |  |  |
| 1672 | Central Operculum L  Globus pallidus L | -38  -24 | 0  -4 | 12  -12 | 0.0202  0.0202 | 5 | 27.8 |
|  |  |  |  |  |  |  |  |
| 1568 | Precentral gyrus R | 62 | -4 | 20 | 0.0202 | 7 | 38.9 |
|  |  |  |  |  |  |  |  |
| 1520 | Anterior insula R | 32 | 16 | 4 | 0.0228 | 6 | 33.3 |
|  |  |  |  |  |  |  |  |
| 1024 | Thalamus L | -8 | -14 | 6 | 0.0217 | 5 | 27.8 |
| 816 | Central operculum R | 40 | 4 | -12 | 0.0215 | 4 | 22.2 |
| 752 | Thalamus R | 10 | -16 | 8 | 0.0177 | 3 | 16.7 |
| 688 | Caudate R | 10  15 | 6  16 | -4  0 | 0.0190  0.0121 | 3 | 16.7 |

^1^ R = right, L = left R = right, L = left

^2^ Total number of experiments for analysis = 18

^3^ All P-values = <.001.

^4^ Using the anterior commissure as the origin of the MNI coordinate system x = from left to right, y = from posterior to anterior, z = from inferior to superior.

Supplementary Table 2. Locations (MNI) of significant clusters from the contrast sucrose minus control (including ROI experiments, sensitivity analysis after removal of Eiler et al. 2018), from an ALE meta-analysis of sweet taste in human adults

| Cluster size (mm^3^) | Brain Region | Peak voxel coordinates | | | ALE value | No of contributing experiments | |
| --- | --- | --- | --- | --- | --- | --- | --- |
|  |  | x | y | z |  | *n* | % |
| 4480 | Mid insula L  Frontal operculum L | -36  -36 | -4  14 | 12  8 | 0.0334  0.0270 | 12 | 70.6 |
| 3256 | Central Operculum R  Frontal operculum R | 40  46 | -6  10 | 14  6 | 0.0253  0.0162 | 13 | 76.47 |
|  |  |  |  |  |  |  |  |
| 2770 | Central operculum L  Precentral gyrus L | -54  -58 | -12  2 | 12  24 | 0.0226  0.0198 | 8 | 47.1 |
|  |  |  |  |  |  |  |  |
| 1248 | Precentral gyrus R | 62 | -4 | 20 | 0.0201 | 5 | 29.4 |
|  |  |  |  |  |  |  |  |
| 1192 | Thalamus L | -8 | -14 | 6 | 0.0217 | 5 | 29.4 |
|  |  |  |  |  |  |  |  |
| 1160 | Anterior insula R | 32 | 16 | 4 | 0.0222 | 4 | 23.5 |
|  |  |  |  |  |  |  |  |
| 984 | Thalamus R | 10 | -16 | 8 | 0.0177 | 4 | 23.5 |

^1^ R = right, L = left R = right, L = left

^2^ Total number of experiments for analysis = 17

^3^ All P-values = <.001.

^4^ Using the anterior commissure as the origin of the MNI coordinate system x = from left to right, y = from posterior to anterior, z = from inferior to superior.

*Significant ALE clusters for the caloric sweetener minus control contrast, including ROI studies.*

When we included the additional 5 ROI studies from the sucrose–control ALE, we observed 11 significant clusters (Supplementary Table 3). These clusters related to: right central operculum/anterior insula/frontal operculum, left insula/frontal operculum, left central operculum and precentral gyrus, right precentral gyrus, left mid insula/globus pallidus, right caudate, left thalamus, right mid insula, left caudate, right anterior cingulate/juxtapositional lobule, and right thalamus.

*Sensitivity analysis:*

After removal of Eiler et al. (1) we observed 7 significant clusters. Four clusters evident in the overall analysis disappeared: right caudate, left caudate, left mid insula/globus pallidus, and right mid insula (Supplementary Table 4).

Supplementary Table 3. Locations (MNI) of significant clusters from the contrast caloric sweeteners minus control (including ROI experiments), from an ALE meta-analysis of sweet taste in human adults

| Cluster size (mm^3^) | Brain Region | Peak voxel coordinates | | | ALE value | No of contributing experiments | |
| --- | --- | --- | --- | --- | --- | --- | --- |
|  |  | x | y | z |  | *n* | % |
| 5728 | Central operculum R | 40 | -6 | 12 | 0.0296 | 18 | 85.7 |
|  | Anterior insula R  Frontal operculum R | 32  48 | 16  10 | 4  6 | 0.0260  0.0178 |  |  |
|  |  |  |  |  |  |  |  |
| 5136 | Insula L  Frontal operculum L | -36  -34 | -4  14 | 10  8 | 0.0357  0.0286 | 15 | 71.4 |
| 2568 | Central operculum L  Precentral gyrus L | -54  -58 | -12  2 | 12  24 | 0.0226  0.0202 | 9 | 42.9 |
|  |  |  |  |  |  |  |  |
| 1760 | Precentral gyrus R | 62 | -4 | 20 | 0.0210 | 7 | 33.3 |
|  |  |  |  |  |  |  |  |
| 1584 | Mid insula L  Globus pallidus L | -38  -24 | 0  -4 | 12  -12 | 0.0203  0.0202 | 5 | 23.8 |
| 1032 | Caudate R  Caudate R | 10  14 | 6  18 | -4  -2 | 0.0191  0.0173 | 5 | 23.8 |
|  |  |  |  |  |  |  |  |
| 968 | Thalamus L | -8 | -14 | 6 | 0.0217 | 4 | 19.0 |
| 896 | Mid insula R | 40 | 4 | -12 | 0.0221 | 4 | 19.0 |
| 704 | Caudate L | -12 | 10 | -2 | 0.0164 | 3 | 14.3 |
| 704 | Anterior cingulate R  Juxtapositional lobule R | 2  8 | 10  6 | 38  48 | 0.0156  0.0148 | 6 | 28.6 |
| 672 | Thalamus R | 10 | -16 | 8 | 0.0170 | 3 | 14.3 |

^1^ R = right, L = left R = right, L = left

^2^ Total number of experiments for analysis = 21

^3^ All P-values = <.001.

^4^ Using the anterior commissure as the origin of the MNI coordinate system x = from left to right, y = from posterior to anterior, z = from inferior to superior.

Supplementary Table 4. Locations (MNI) of significant clusters from the contrast caloric sweeteners minus control (including ROI experiments, sensitivity analysis after removal of Eiler et al. 2018), from an ALE meta-analysis of sweet taste in human adults

| Cluster size (mm^3^) | Brain Region | Peak voxel coordinates | | | ALE value | No of contributing experiments | |
| --- | --- | --- | --- | --- | --- | --- | --- |
|  |  | x | y | z |  | *n* | % |
| 5304 | Insula R | 32 | 16 | 4 | 0.0254 | 16 | 76.2 |
|  | Central operculum R  Frontal operculum R | 40  48 | -6  10 | 14  6 | 0.0254  0.0178 |  |  |
|  |  |  |  |  |  |  |  |
| 4792 | Insula L  Frontal operculum L | -36  -36 | -4  14 | 12  8 | 0.0334  0.0273 | 12 | 57.1 |
| 2616 | Central operculum L  Precentral gyrus L | -54  -58 | -12  2 | 12  24 | 0.0226  0.0202 | 8 | 38.1 |
|  |  |  |  |  |  |  |  |
| 1464 | Precentral gyrus R | 62 | -4 | 20 | 0.0210 | 5 | 23.8 |
|  |  |  |  |  |  |  |  |
| 1088 | Thalamus L | -8 | -14 | 6 | 0.0217 | 5 | 23.8 |
| 848 | Thalamus R | 10 | -16 | 8 | 0.0177 | 4 | 19.0 |
|  |  |  |  |  |  |  |  |
| 840 | Anterior cingulate | 2 | 10 | 38 | 0.0156 | 6 | 28.6 |
|  | Juxtapositional lobule cortex | 8 | 6 | 48 | 0.0148 |  |  |

^1^ R = right, L = left R = right, L = left

^2^ Total number of experiments for primary analysis = 20

^3^ All P-values = <.001.

^4^ Using the anterior commissure as the origin of the MNI coordinate system x = from left to right, y = from posterior to anterior, z = from inferior to superior.

Supplementary Table 5. Study details of ROI studies meeting inclusion criteria, and non-caloric sweetener studies found from a systematic review of neuroimaging sweet taste in human adults ^1^

| Study [Supplementary reference] | Contrast | Concentration | Pleasantness/intensity | Hunger state | *n* (male) | Age (years) | Hand | BMI | Foci/clusters | Statistical correction | Whole brain/mask |
| --- | --- | --- | --- | --- | --- | --- | --- | --- | --- | --- | --- |

| **Sucrose ROI experiments** |  |  |  |  |  |  |  |  |  |  |  |
| --- | --- | --- | --- | --- | --- | --- | --- | --- | --- | --- | --- |
| de Araujo et al. (2) | Sucrose minus tasteless solution | 0.5 M | Midpoint of response intensity functions | NA | 11 (6) | NA | R | Not reported | 8: Caudomedial OFC, anterior insula, operculum, ventral forebrain, amygdala, anterior cingulate cortex | uncorrected p<0.0001 | ROI |
|  |  |  |  |  |  |  |  |  |  |  |  |
| Rudenga & Small (3) | Sucrose minus tasteless solution | “strong” | Solution rated as “strong” (33.0±13.3), pleasantness varied widely (7.5±50.0), (out of 100) | Neither hungry nor full | 26 (10) | 19-38 | NA | 19-28 | 2: Rolandic operculum, insula, striatum | p<0.05, k≤5, FDR-corrected across the entire brain | ROI |
|  |  |  |  |  |  |  |  |  |  |  |  |
| Orbendorfer et al. (4) | Sucrose minus baseline | 0.29 M |  | Standardized breakfast | 14 (0) |  | NA | 22.6 ± 1.5 | 8: Insula, thalamus, middle frontal gyrus, supragenual anterior cingulate cortex | Monte Carlo | ROI |
|  |  |  |  |  |  |  |  |  |  |  |  |
| Rudenga & Small. (5) | Sucrose (strong sweet + weak sweet) minus tasteless solution | Strong sweet = 1.45 ± 0.4 M. weak sweet = 0.086 ± 0.058 M | Intensity and pleasantness categorised by likers and dislikers | NA | 30 (12) | 24.5 ± 5.8 | 27 R | 23.4 ± 2.6 | 11: Putamen, insula, ventral striatum, thalamus, striatum | multiple thresholds for apriori hypotheses | ROI |
|  |  |  |  |  |  |  |  |  |  |  |  |
| Monteleoni et al. (6) | Sucrose minus water | 0.29 M | Pleasantness rating 3.27 ± 1.19 (out of 10) | NA | 20 (0) | 27.1 ± 4.7 | NA | 21 ± 1.5 | 13: Middle cingulate cortex, anterior insula, inferior insula, amygdala, posterior insula, postcentral gyrus, brainstem | Monte Carlo | ROI |
|  |  |  |  |  |  |  |  |  |  |  |  |
| **Totals** | **5 experiments** |  |  |  | **101** |  |  |  | **42** |  |  |
|  |  |  |  |  |  |  |  |  |  |  |  |
| **Experiments with sugars other than sucrose** |  |  |  |  |  |  |  |  |  |  |  |

| O’Doherty et al. (7) | Glucose minus tasteless solution | 1.0 M | Pleasantness (from 2 very pleasant, 0 neutral, -2 very unpleasant) = 1.06 ± 0.17 | NA | 8 (5) | 24.5 (18-35) | NA | Not reported | 1: Dorsal frontal operculum | Uncorrected p<0.001 |
| --- | --- | --- | --- | --- | --- | --- | --- | --- | --- | --- |

|  |  |  |  |  |  |  |  |  |  |  |  |
| --- | --- | --- | --- | --- | --- | --- | --- | --- | --- | --- | --- |
| **Totals** | **1 experiment** |  |  |  | **8** |  |  |  | **1** |  |  |
|  |  |  |  |  |  |  |  |  |  |  |  |
|  |  |  |  |  |  |  |  |  |  |  |  |

| **Non-caloric sweetener experiments** | |  |  |  |  |  |  |  |  |  |  |  |
| --- | --- | --- | --- | --- | --- | --- | --- | --- | --- | --- | --- | --- |
|  |  | |  |  |  |  |  |  |  |  |  |  |
| Chambers et al. (8) | Saccharin minus tasteless solution | | 0.27 M | Sweetness: 39±18, pleasantness = 54 ± 12 (out of 100) | Overnight fast | 7 (7) | 23±3 | R | 22.2 ± 1 | 2: Insula/operculum, DLPFC | Monte Carlo | Whole Brain |
|  |  | |  |  |  |  |  |  |  |  |  |  |
| Green & Murphy (9) | Saccharin minus water | | 0.014 M | Mean pleasantness rating: 54.6±10.0, mean intensity rating: 38.6 ± 23.8 (out of 100) | Fasted for 12 hours | 12 (5) | 23.0 ± 2.3 | R | 25.03 ± 5.6 | 22: cerebellum, postcetral gyrus/BA1, precuneus, cerebellum, precentral gyrus, thalamus/medial dorsal nucleus, Insula, thalamus, ostcentral gyrus, BA13, cingulate gyrus, inferior frontal gyrus, middle frontal gyrus, paracentral lobule, superior parietal lobule | Monte Carlo | Whole Brain |
|  |  | |  |  |  |  |  |  |  |  |  |  |
| Haase et al. (10) | Saccharin minus water | | 0.028 M | Pleasantness not discussed | Fasted for 12 hours | 18 (9) | 20.7 ± 1.0 | NA | Males = 24.4, females = 23.03 | 3: Cuneus, lingual gyrus, thalamus | Monte Carlo | Whole Brain |
|  |  | |  |  |  |  |  |  |  |  |  |  |
| Frank et al. (11) | Sucralose main effect | | 0.0032 M | Pleasantness slopes were calculated which predicted left insula activity | Sated following breakfast | 12 (0) | 27±6 | NA | 22 ± 2 | 3: Frontal operculum/anterior insula/claustrum, right superior frontal gyrus | Main effect statistical maps were thresholded at p<0.005 and  (minimum of 16 contiguous voxels) | Whole Brain |
|  |  | |  |  |  |  |  |  |  |  |  |  |
| Oberndorfer et al. (4) | Sucralose minus baseline | | Rated equivalent taste to 0.29 M sucrose |  | Standardized breakfast | 14 |  | NA | 22.6 ± 1.5 | 8: Insula, thalamus, middle frontal gyrus, supragenual anterior cingulate cortex, midbrain | Monte Carlo | ROI |
|  |  | |  |  |  |  |  |  |  |  |  |  |
| **Totals** | **5 experiments** | |  |  |  | **63** |  |  |  | **38** |  |  |

^1^ BA = Broca’s Area, BMI = Body Mass Index, DLPFC = Dorsolateral Prefrontal Cortex, FDR = False Discovery Rate, k = cluster size in units of contiguous clusters, MNI = Montreal Neuroimaging Institute M=Molar, OFC = Orbitofrontal Cortex, ROI = Region of Interest.

^2^ Values are means ± SDs or ranges unless otherwise indicated

Supplementary References

1. Eiler II WJ, Dzemidzic M, Soeurt CM, Carron CR, Oberlin BG, Considine RV, et al. Family history of alcoholism and the human brain response to oral sucrose. NeuroImage: Clinical. 2018;17:1036-46.

2. De Araujo IE, Rolls ET, Kringelbach ML, McGlone F, Phillips N. Taste‐olfactory convergence, and the representation of the pleasantness of flavour, in the human brain. European Journal of Neuroscience. 2003;18(7):2059-68.

3. Rudenga KJ, Small DM. Amygdala response to sucrose consumption is inversely related to artificial sweetener use. Appetite. 2012;58(2):504-7.

4. Oberndorfer TA, Frank GKW, Simmons AN, Wagner A, McCurdy D, Fudge JL, et al. Altered insula response to sweet taste processing after recovery from anorexia and bulimia nervosa. The American Journal of Psychiatry. 2013;170(10):1143-51.

5. Rudenga KJ, Small DM. Ventromedial prefrontal cortex response to concentrated sucrose reflects liking rather than sweet quality coding. Chemical Senses. 2013;38(7):585-94.

6. Monteleone AM, Monteleone P, Esposito F, Prinster A, Volpe U, Cantone E, et al. Altered processing of rewarding and aversive basic taste stimuli in symptomatic women with anorexia nervosa and bulimia nervosa: An fMRI study. Journal of psychiatric research. 2017;90:94-101.

7. O'Doherty JP, Deichmann R, Critchley HD, Dolan RJ. Neural responses during anticipation of a primary taste reward. Neuron. 2002 Feb 28;33(5):815-26.

8. Chambers E, Bridge M, Jones D. Carbohydrate sensing in the human mouth: effects on exercise performance and brain activity. The Journal of physiology. 2009;587(8):1779-94.

9. Green E, Murphy C. Altered processing of sweet taste in the brain of diet soda drinkers. Physiology & behavior. 2012;107(4):560-7.

10. Haase L, Cerf-Ducastel B, Murphy C. Cortical activation in response to pure taste stimuli during the physiological states of hunger and satiety. Neuroimage. 2009;44(3):1008-21.

11. Frank GK, Oberndorfer TA, Simmons AN, Paulus MP, Fudge JL, Yang TT, et al. Sucrose activates human taste pathways differently from artificial sweetener. Neuroimage. 2008;39(4):1559-69.
